# Novel bone morphogenetic protein 15 (*BMP15*) gene variants implicated in premature ovarian insufficiency

**DOI:** 10.1186/s12958-022-00913-6

**Published:** 2022-03-01

**Authors:** Fatemeh Afkhami, Shirin Shahbazi, Laya Farzadi, Shahla Danaei

**Affiliations:** 1 Department of Medical Genetics, Faculty of Medical Sciences, Tarbiat Modares University, Tehran, Iran; 2 Department of Obstetrics and Gynecology, School of Medicine, Tabriz University of Medical Sciences, Tabriz, Iran; 3 Gynecology Departments, Eastern Azerbaijan ACECR ART Center, Eastern Azerbaijan Branch of ACECR, Tabriz, Iran

**Keywords:** POI, BMP15, Gene mutation, *In silico* analysis

## Abstract

**Background:**

Bone morphogenetic protein 15 (*BMP15*) is expressed in oocytes and plays a crucial role in the reproduction of mono-ovulating species. In humans, *BMP15* gene mutations lead to imperfect protein function and premature ovarian insufficiency. Here we investigated the *BMP15* gene variants in a population of Iranian women with premature ovarian insufficiency. We conducted predictive bioinformatics analysis to further study the outcomes of *BMP15* gene alterations.

**Methods:**

Twenty-four well-diagnosed premature ovarian insufficiency cases with normal karyotype participated in this study. The entire coding sequence and exon-intron junctions of the *BMP15* gene were analyzed by direct sequencing. *In-silico* analysis was applied using various pipelines integrated into the Ensembl Variant Effect Predictor online tool. The clinical interpretation was performed based on the approved guidelines.

**Results:**

By gene screening of *BMP15*, we discovered p.N103K, p.A180T, and p.M184T heterozygous variants in 3 unrelated patients. The p.N103K and p.M184T were not annotated on gnomAD, 1000 Genome and/or dbSNP. These mutations were not identified in 800 Iranians whole-exome sequencing that is recorded on Iranom database. We identified the p.N103K variant in a patient with secondary amenorrhea at the age of 17, elevated FSH and atrophic ovaries. The p.M184T was detected in a sporadic case with atrophic ovaries and very high FSH who developed secondary amenorrhea at the age of 31.

**Conclusions:**

Here we newly identified p.N103K and p.M184T mutation in the *BMP15* gene associated with idiopathic premature ovarian insufficiency. Both mutations have occurred in the prodomain region of protein. Despite prodomain cleavage through dimerization, it is actively involved in the mature protein function. Further studies elucidating the roles of prodomain would lead to a better understanding of the disease pathogenesis.

## Background

Premature Ovarian Insufficiency (POI) also called premature ovarian failure (POF) is considered by loss of ovarian activity before the age of 40. It can be distinguished as primary amenorrhea with delayed menarche, secondary amenorrhea and oligomenorrhea over 4 months. POI is diagnosed by an increase in follicle-stimulating hormone (FSH) of higher than 25mIU/ml, repeated twice over 4 weeks [1]. Antimullerian hormone (AMH) is produced by the granulosa cells of growing follicles and is of considerable value in the timely detection of POI. Ovarian reserve can be detected by transvaginal ovarian ultrasound and estimated by AMH levels in POI cases [2]. The prevalence of POI was mainly considered 1 -1.5%. However, a recent meta-analysis reported a global prevalence of 3.7% with a higher rate in medium and low developed countries [3].

In the aetiology of POI, a wide range of explanations have been considered including involvement of infectious, autoimmune, iatrogenic or genetic factors. Among them, genetic aberrations account for approximately 20–25% of cases. FMR1 permutation is the most common genetic abnormality in POI, followed by bone morphogenetic protein 15 (*BMP15*) gene defects [4]. *BMP15* gene is located on Xp11.2 and encodes a 392- amino acid protein of transforming growth factor-β superfamily (TGF-β). The protein is composed of the signal peptide, prodomain and transforming growth factor β- like (TGFβ-like) domain [5]. BMP15 is expressed in oocytes and acts synergically with growth differentiation factor 9 (GDF9) towards the regulation of folliculogenesis [6]. Using a mouse model, it has been shown that BMP15 promote follicular development along with FSH [7]. In vitro study revealed that the simultaneous addition of GDF9 and BMP15 to the culture leads to improvement of the human primordial follicles activation [8].

BMP15 peptides either form homodimers or bind to GDF9 and make heterodimers called cumulin. Dimerization occurs via the TGFβ-like domain. A furin-like protease cleaves prodomain upon protein maturation, however, prodomain remains associated with secreted mature dimer. BMP15 and cumulin control the migration and proliferation of primordial germ cells through separate signal pathways [9, 10].

Pathogenic mutations can disrupt the transcription/translation of BMP15 protein or weaken the interaction with GDF9 [11]. Preliminary studies on knockout models have shown that *GDF-9* deficiency led to impaired folliculogenesis and infertility, while a null mutation in the *BMP-15* gene caused only reduced fertility in female mice [12]. Subsequent studies suggested differences in BMP15 function between mice and other mammals. A study aiming to identify factors regulating ovulation rate in sheep found that heterozygous carriers of *BMP15* mutation had increased ovulation while homozygous were infertile [13]. Additional evidence from the porcine knockdown model confirmed that the role of BMP15 in single-ovulatory species is more pronounced than in multi-ovulatory species [14]. This difference is explained in the balance of GDF9 and BMP15. The dominance of GDF9 signaling leads to multiple ovulations and high fertility, whereas the production of BMP15 modulate pri-mordial germ cell sensitivity to gonadotropins and leads to single ovulation, low fertility and appropriate ovarian reserve [15].

Considering the importance of BMP15 in the pathogenesis of POI, we aimed to investigate the related mutations in a population of affected Iranian women. We further conducted predictive bioinformatics analysis in a comprehensive manner, to evaluate *BMP15* gene alterations linked to POI.

## Methods

### Patients

This study was approved by the Ethics Committee of Tarbiat Modares University, Tehran, Iran (IR.MODARES.REC.1399.006). The clinical interpretation was performed based on the European society of human reproduction and embryology (ESHRE) guidelines [2]. All participants delivered written informed consent before participating in the study. Physical examination, blood testing and sonography were performed as part of routine clinical workup by the experienced gynecologists. Twenty four normal-karyotype women with idiopathic secondary amenorrhea before the age of 40 were entered into our study. Based on the genetic counselling, patients were further allocated into sporadic and familial groups. Having at least two serum FSH levels higher than 25mIU/ml was the main inclusion criteria. The exclusion criteria comprised any of the following: chromosomal aberrations including FMR1 premutation, personal or family history of autoimmune diseases, ovarian surgery, radiotherapy or chemotherapy. To rule out autoimmune disease, related serological tests containing thyroid peroxidase antibody, anti-tissue transglutaminase IgG and thyroglobulin antibody were checked.

### DNA extraction and PCR-Sequencing

Genomic DNA was extracted from whole blood samples using the salting-out method. The purity and concentration of the extracted DNA were measured by spectrophotometry. Specific primers were designed for two exons of BMP15 using Oligo explorer and Primer Express software as follows: exon1F;GTTGTGGAGCCAGGATGCAG, exon1R;AAGCCTGACAGTAAACCCACC, exon2F;AATTTTAGGGCTGATTATAGC, exon2R;TTGGTACAGGATTACTTGCAG.

To perform the PCR reaction, 2X PCR master mix (Amplicon, Pishgam, Iran) containing an ultimate concentration of 1.5 mM MgCl2 was used in a final volume of 50 µl. The PCR program included 5 min initial priming at 95 °C and then 30 cycles with 30 s at 95 °C, 30 s at 59 °C, 30 s at 72 °C with a final extension of 72 °C for 10 min. The PCR products were electrophoresed on 1.5% agarose gel along with the 100 base pairs DNA marker. Bi-directional Sanger sequencing was performed on all samples across two coding exons and flanking intronic sequences. Sanger sequencing results were interpreted using the Chromas software version 2.01.

### In silico analysis

The probable pathogenic effect of the amino acid changes was detected by SIFT, PolyPhen, etc., which were integrated into the Ensembl Variant Effect Predictor (VEP) https://asia.ensembl.org/Homo_sapiens/Tools/VEP?db=core. The prevalence of the variants was checked on the Iranome website http://www.iranome.ir/. The Iranome browser represents whole-exome sequencing data of 800 healthy individuals from eight Iranian major ethnic groups with the approximate 1:1 Female/Male ratio. All samples were selected from individuals who were > 30 years old to reduce the bias of late-onset Mendelian disorders. The mean age at blood draw was 50.61 ± 9.33, and the age range 30–84 years old in the whole project [16].

Structural modeling of BMP15 wild-type and missense variants were computed by the SWISS-MODEL servers. The protein models were assessed using the Ramachandran plot from the MolProbity program.

### Statistical analyses

The collected clinical data were statistically analyzed using SPSS version 24 with a significance level chosen at 5%. Correlation studies of the quantitative and qualitative data were performed by ANOVA and Chi-square tests, respectively.

## Results

### Clinical status of patients

All of the patients had spontaneously entered menarche and none of the patients was exhibited primary amenorrhea. The mean age at menarche was calculated at 13.12 ± 1.56 years old. The mean age at POI diagnosis was 26.84 ± 9.07 years old ranged from 12 to 39. Median and mean levels of serum FSH were 40.8 and 75.92 mIU/ml, respectively. The analyses of AMH serum levels were available for 19 out of 24 patients and the majority of them were below 0.02ng/ml. The sonographic evaluation revealed that 79.16% of the patients had atrophic ovaries and 20.83% had normal ovarian pattern. Patients’ interviews and genetic counselling disclosed that 6 out of 24 cases had a family history of menstrual irregularities, infertility and/or secondary amenorrhea.

### BMP15 genotyping

**Fig. 1:**
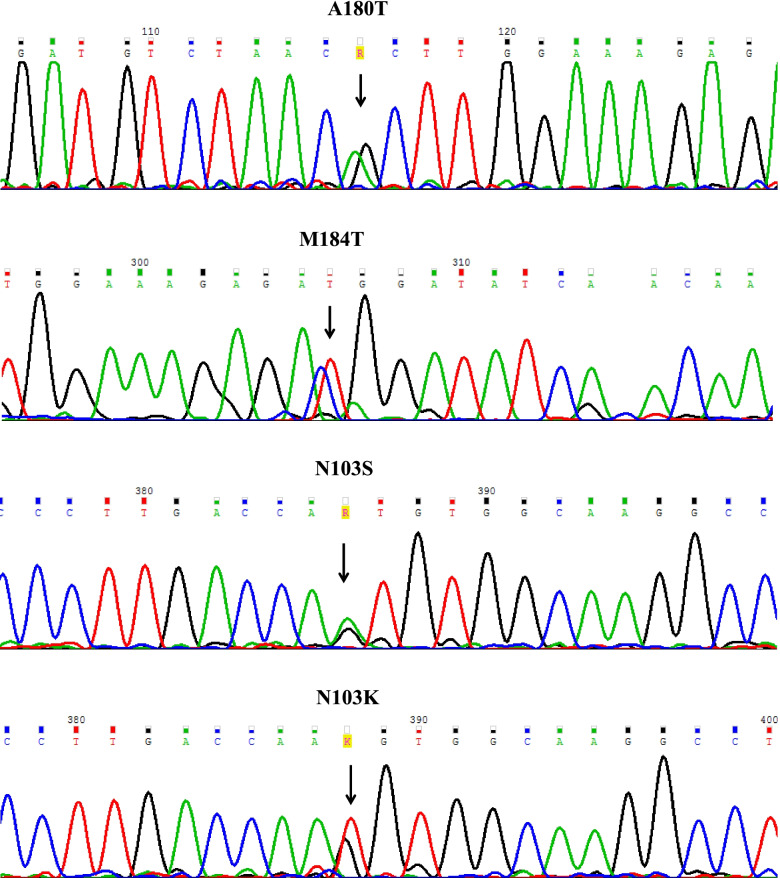
Electropherogram analysis of mutations and SNP alleles in unrelated POI patients

As shown in Fig. 1, by the PCR-sequencing analysis of *BMP15*, we discovered p.N103K, p.A180T, and p.M184T heterozygous variants in 3 unrelated patients. This is the first report of the link between p.N103K and p.M184T to the POI pathogenesis.

The patient carrier of p.N103K variant entered menarche at the age of 16 and secondary amenorrhea happened when she was at the age of 17. As specified in Table 1, elevated FSH levels and a low normal range of AMH were detected in this patient. The sonographic evaluation exhibited an atrophic ovaries pattern. She was born from a consanguineous background without any indication of POI or related conditions in her family members.

**Table 1:** Identified variants and clinical characteristics of the patients according to the ESHRE guideline

Variant	Current Age (yrs.)	Age at Menarche (yrs.)	Patterns of menses at Diagnosis	Age at Diagnosis (yrs.)	Sonographic finding at Diagnosis	FSH (mIU/mL) at Diagnosis	AMH (ng/ml) at Diagnosis	Maternal Parity
p.N103S	29	13	Secondary Amenorrhea	17	Atrophic Ovaries	135	0.01	-
p.N103K	20	16	Secondary Amenorrhea	17	Atrophic Ovaries	40	0.82	-
p.A180T	42	13	Irregular menses (Oligomenorrhea more than 18 months)	39	Normal Ovaries	30	0.03	One Child
p.M184T	33	16	Secondary Amenorrhea	31	Atrophic Ovaries	100	0.01	One Child

p.M184T was detected in a sporadic POI patient who was prematurely menopaused at the age of 31. High FSH levels of 100mIU/ml and very low levels of AMH were detected by hormonal profiling. Ultrasound examination showed an atrophic pattern in both ovaries. Detailed family history revealed that the parents were not relatives and there was no history of POI and/or related disorders among her relatives.

We identified p.A180T in a 42-years-old patient who was a descendant of consanguineous marriage with a familial history of POI. She had been diagnosed at age of 39 following a history of oligomenorrhea for more than 18 months. The hormonal assessment showed high FSH and very low AMH levels. The sonographic evaluation indicated normal ovarian status (Table 1).

We also detected two known variants of *BMP15;* c.-9 C > G and p.N103S [17]. Previous studies from Syria and India reported the p.N103S, in both cases and controls [18, 19]. This variant is mentioned in the ClinVar database as a benign variant causing ovarian dysgenesis. The annotation of its corresponding allele, c.308 A > G showed an allele frequency of 0.057 and 0.028 in gnomAD and 1000 Genome, respectively. Iranome browser specified a high c.308 A > G allele frequency of 0.075 in the Iranian population. In our study 1 out of 24 patients (1/48 alleles) was the carrier of this SNP variant.

### rs3810682, c.-9 C > G

The 5`-UTR variant, c.-9 C > G, was detected in 29.16% of the patients, all in heterozygous status including 2 compound heterozygous with p.A180T and p.N103S. Statistical analysis was performed to calculate the correlation between c.-9 C > G SNP and clinicopathological parameters of the patients. Table 2 shows a statistically significant association between c.-9 C > G SNP and patients FSH levels (*p value* = 0.02).

**Table 2:** Correlation between the − 9G polymorphism and clinicopathological parameters of the patients,

Clinical Indicators	-9G Polymorphism		P	chi-square
	Yes (%)	No (%)		
Age at menopause (yrs.)			.47	0.50
*<30*	3 (42.9)	10 (58.8)		
*≥30*	4 (57.1)	7 (41.2)		
Age at menarche (yrs.)			.56	0.33
*<13*	5 (71.4)	10 (58.8)		
*≥13*	2 (28.6)	7 (41.2)		
FSH levels (mIU/ml)			**.02**	4.88
*<40*	3 (42.9)	1 (5.9)		
*≥40*	4 (57.1)	16 (94.1)		
AMH levels (ng/ml)			.07	3.26
*<.02*	3 (50)	10 (76.9)		
*≥.02*	3 (50)	3(23.1)		
Sonographic findings			.61	0.25
*Normal Ovaries*	1 (14.3)	4 (23.5)		
*Atrophic Ovaries*	6 (85.7)	13 (76.5)		

### In-silico analysis

Further *in-silico* analysis was conducted to search the effects of the newly identified p.N103K and p.M184T on protein biogenesis and function. Table 3 lists the *in-silico* predicted values of these mutations in comparison to previously reported nonsynonymous missense alterations of *BMP15.* The indicated data were extracted from the Ensembl VEP online tool.

**Table 3:** Variants annotation, allele frequencies and *in-silico* prediction of nonsynonymous missense alterations of *BMP15*

Reference	Position/ Substitution	Amino acids	Existing variant	AF	gnomAD AF	Iranome AF	SIFT	PolyPhen	CADD PHRED
[20]	13 A/C 50,910,796 **A**GT/**C**GT	S5R	rs113099187 CM0910176	0.0358	0.009278	-	0.01 Deleterious	0.368 Benign	13.19
[19]	181 C/T 50,910,964 **C**GG/**T**GG	R61W	rs144392417 CM061653	-	-	-	0.24 Tolerated	0 Benign	9.513
[21]	202 C/T 50,910,985 **C**GG/**T**GG	R68W	rs104894763 CM061661	0.0005	0.0006997	-	0 Deleterious	0.877 Possibly damaging	22.4
[19]	226 C/T 50,911,009 **C**GT/**T**GT	R76C	rs104894766 CM061662 COSV53141666 COSV99399454	0.0013	0.0004129	0.005625	0 Deleterious	0.959 Probably damaging	22.8
[19]	227G/A 50,911,010 C**G**T/C**A**T	R76H	rs1557279925 CM061656 COSV53140125	-	1.977e-05	-	0.06 Tolerated	0.959 Probably damaging	20.8
[22]	242 A/G 50,911,025 C**A**T/C**G**T	H81R	rs781801740	-	-	-	0.47 Tolerated	0.001 Benign	0.010
[19]	308 A/G 50,911,091 A**A**T/A**G**T	N103S	rs41308602 CM1513124 COSV53140249	0.0283	0.05707	0.07500	0.3 Tolerated	0 Benign	0.190
	**309T/G** **50,911,092** AA**T/**AA**G**	**N103K**	**-**	**-**	**-**	**-**	0.35 Tolerated	0.006 Benign	3.452
[23]	G/C 50,915,834 **G**TT/**C**TT	V136L	rs1387861526	-	5.465e-06	-	0.02 Deleterious	0.292 Benign	15.84
[20]	413G/A 50,915,841 C**G**C/C**A**C	R138H	rs371418883 CM092909 COSV53139787	-	3.281e-05	-	0.56 Tolerated	0.854 Possibly damaging	9.814
[20]	443T/C 50,915,871 C**T**C/C**C**C	L148P	rs114823607 CM061658	0.0109	0.003174	-	0 Deleterious	0.987 Probably damaging	23.0
[21]	538G/A 50,915,966 **G**CT/**A**CT	A180T	rs104894767 CM061654 CX062295 COSV53141311	0.0032	0.01001	0.006875	0.33 Tolerated	0.007 Benign	0.059
	**551T/C** **50,915,979** A**T**G**/**A**C**G	**M184T**	**-**	**-**	**-**	**-**	0.13 Tolerated	0.003 Benign	7.245
[22]	581T/C 50,916,009 T**T**C/T**C**C	F194S	rs141218518	0.0008	0.002045	0.001250	0.09 Tolerated	0.003 Benign	13.02
[19]	588T/A 50,916,016 AA**T**/AA**A**	N196K	CM061651	-	-	-	0.07 Tolerated	0.027 Benign	6.408
[22]	595 G/A 50,916,023 **G**GA/**A**GA	G199R	rs782378869 COSV53142031	-	2.181e-05	-	0.54 Tolerated	0.018 Benign	0.415
[24]	598 C/T 50,916,026 **C**AC/**T**AC	H200Y	rs202165852	0.0003	0.0002781	-	0.53 Tolerated	0.001 Benign	2.424
[19]	617G/A 50,916,045 C**G**T/C**A**T	R206H	rs782516193 CM061657	-	6.543e-05	-	0.46 Tolerated	0.003 Benign	0.734
[19]	661 T/C 50,916,089 **T**GG/**C**GG	W221R	rs375284458 CM061659	0.0003	0.000398	-	0.13 Tolerated	0.174 Benign	11.79
[25]	704 A/G 50,916,132 T**A**T/T**G**T	Y235C	rs104894765 CM041254	-	-	-	0 Deleterious	0.95 Probably damaging	22.7
[24]	985 C/T 50,916,413 **C**GC/**T**GC	R329C	rs782375794 CM100080 COSV99399521	-	1.653e-05	-	0.02 Deleterious	0.916 Probably damaging	22.5
[26]	986G/A 50,916,414 C**G**C/C**A**C	R329H	rs782306478	-	1.1e-05	-	0.57 Tolerated	0.007 Benign	0.095
[27]	1070G/A 50,916,498 T**G**T/T**A**T	C357Y	rs1557280378	-	1.097e-05	-	0 Deleterious	1 Probably damaging	24.3

*AF *Frequency of existing variants in 1000 genomes combined population, *gnomAD AF* Frequency of existing variants in gnomAD exomes combined population, *Iranome AF* Allele frequency in Iranome database

The newly identified p.N103K was not annotated on gnomAD, 1000 Genome or dbSNP. Investigation on the Iranome database revealed that p.N103K did not occur in 800 healthy Iranians. The SIFT and PolyPhen results showed scores of 0.35 and 0.006, respectively. In the same way, the p.M184T new mutation was not annotated in databases. Bioinformatics analysis showed scores of 0.13 and 0.003 by the SIFT and PolyPhen prediction tools, respectively.

**Fig. 2:**
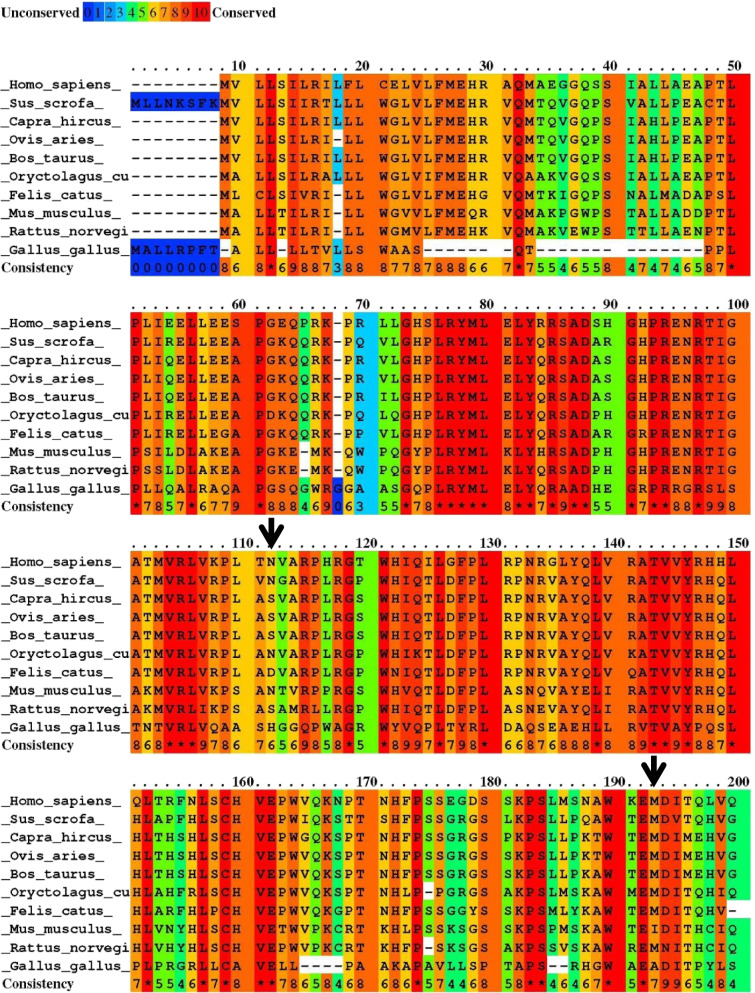
The conservation scores of the first 200 amino acids of BMP15 protein among different species. Blue and red lines show the two side of the spectrum from amino acid insertions or differences with the lowest degree of conservation to sequence identity among different species, respectively. Black arrows indicate the score of N103 and M184 residues, respectively. Analyzed on https://www.ibi.vu.nl/programs/pralinewww/

**Fig. 3:**
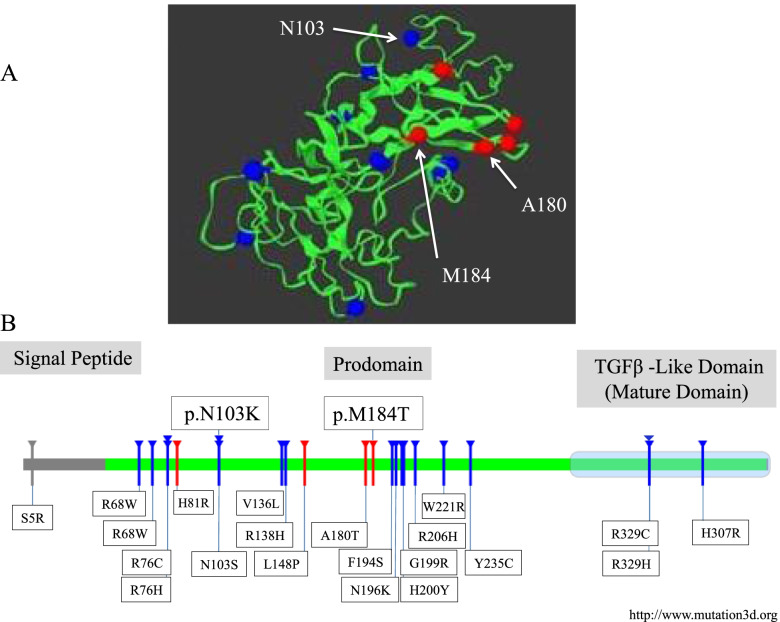
In **A**, the location of N103, A180 and M184 are shown. **B** indicates the location of reported nonsynonymous missense alterations of *BMP15*. Most of the variants are located on prodomain except for p.R329C, p.R329H and p.C357Y which are located on the mature TGFβ-like domain

Figure 2 shows the conserved score of the M184 and N103 residues among different species indicating a higher score for M184. Both mutations are located in the secondary structure elements of the prodomain (Fig. 3B), which are essential for regulating mature protein production, secretion, and activity. M184 and N103 are present in β6 and β1` sheet of the peptide structure, respectively [26]. As shown in Fig. 3 A, M184 and A180 are situated in the same β-sheet of the peptide structure.

### Modelling and Mapping of the wild-type and missense variants

**Fig. 4:**
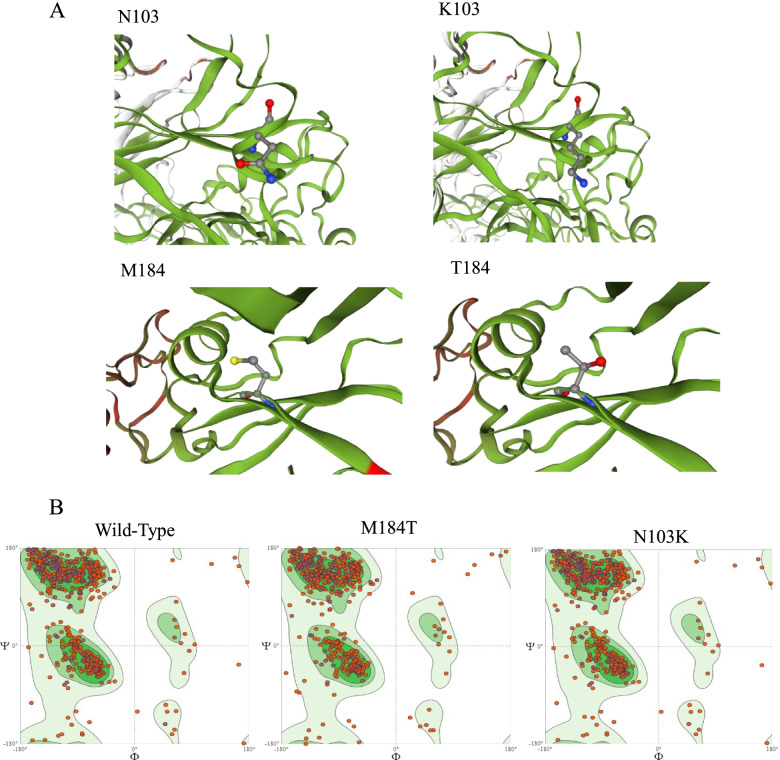
**A** Modeled BMP15 amino acid replacement on the three-dimensional structure. **B** General (no proline or glycine) Ramachandran plots of the BMP15 wild-type, M184T and N103K proteins which were provided by SWISS-MODEL online server. The different colored areas correspond to favored, allowed, generously allowed and sterically disallowed conformations

Figure 4 A shows molecular modeling of wild-type M184 and N103 as compared with mutant T184 and K103, respectively. The results confirmed the localization of amino acids on the protein β-sheet structures. Figure 4B provides Ramachandran plots exhibiting Φ/Ψ backbone dihedral angles of the wild- type and mutant proteins. The wild-type showed 88.4% and 4.03% residues in the favored and outliers regions of the plot. The generated model for M184T was found to have fewer residues in the favored region (87.6%). The Rotamer Outliers was also increased for the M184T (0.79%) vs. wild- type (0.16%) which indicates a decrease in protein stability. The mutant N103K displayed the same configuration as that of the wild-type structure.

### Segregation analysis of the p.A180T

**Fig. 5:**
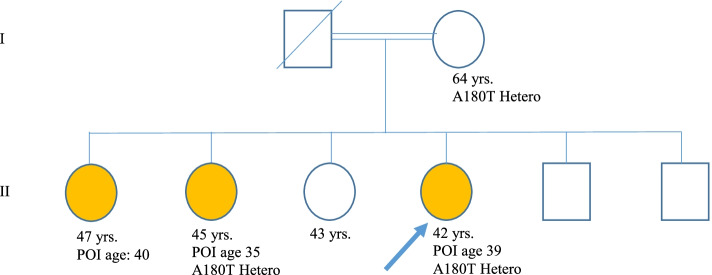
Family pedigree shows the proband (arrow) and the rest of the affected family members with POI presented in colored circles. Genotyping of BMP15 is indicated below the symbols, with the A180T heterozygote variant

As mentioned earlier, p.A180T was found in a patient with a family history of POI. The c.538G˃A missense variant located in exon 2 of *BMP15* is presented in databases as the rs104894767. As illustrated in the family pedigree, two older sisters of the proband were also reached menopause before the age of 40, however, their mother entered physiological menopause at the age of 55. By Sanger sequencing, the c.538G˃A variant was analyzed in the female family members. The obtained results indicated a heterozygote alteration in the 64-years-old mother and one of the affected sisters who was menopaused at 35. The other POI sister with menopause at 40 and the 43-years-old healthy sister with regular menses did not carry the p.A180T variant (Fig. 5).

## Discussion

In this study, we provided the molecular analysis of the *BMP15* gene in a series of unrelated Iranian women with idiopathic POI. Our results revealed two novel variants; p.N103K and p.M184T as well as one known substitution; p.A180T.

With the development of next-generation sequencing, several new genes have been linked to the disease pathogenesis which supports the concept of POI heterogeneity. Investigation of copy number variations led to the identification of microdeletions/microduplications on multiple chromosomal regions. Using high-resolution SNP arrays a 4 MB deletion in Xp11.23-p11.22 was discovered in a patient with secondary amenorrhea at the age of 34. This deletion consists of 65 genes, including the entire *BMP15* [28]. A more recent study reported the deletion of both paternal and maternal copies of *BMP15* in a 16-year-old patient with primary amenorrhea and FSH levels of 141mIU/ml. The deletion expanded 36 KB and contained the entire *BMP15* in the Xp11.22 region. In addition, the study showed intragenic duplication of the tumor protein P63 (*TP63*) gene in two sisters who were also the carrier of *BMP15* p.Y235C [29]. Remarkably, p.Y235C is the first discovered *BMP15* mutation that was reported in 2004 in the heterozygous Italian sisters [25]. The same research group further identified p.R68W and p.A180T alterations in non-familial POI patients in 2006 [21]. In 2009 they repeated the *BMP15* gene screening in 300 patients and found p.R138H and p.L148P in the prodomain region. They also reported p.S5R mutation which is localized in the signal peptide of the nascent protein. By conducting a functional *in vitro* assay in the human granulosa cell line, they disclosed that p.R68W, p.L148P and p.R138H cause the reduction of mature BMP15 protein. While p.A180T or p.S5R displayed no deleterious impact on protein secretion or function [20]. However, in 2017, Patiño et al. estimated four-fold lower activity of p.A180T compared to wild-type BMP15. They evaluated the expression and activity of 10 *BMP15* variants and showed that these nucleotide changes could reduce mature peptide production, activity or synergy with GDF9 [26].

The obtained results regarding p.A180T remained conflicting, as in several studies it was only detected in patients [11, 19], while in others, both in patients and controls [22, 30]. Based on the Iranome database, the allele frequency of the p.A180T in the Iranian healthy population is 0.006. In our study, we found the p.A180T variant in a POI patient who was born from a consanguineous marriage with a family history of POI. Segregation analysis confirmed that the p.A180T could not be the causative mutation in this family. Modifier genes and SNPs should be considered as the influential factors in the genotype/phenotype correlation.

We further identified p.N103K missense heterozygote transition. This new *BMP15* mutation was detected in the offspring of first-cousin parents without any family history of POI. Our next newly identified missense heterozygote substitution; p.M184T was identified in a sporadic POI case exhibiting high FSH levels. Since p.N103K and p.M184T are not annotated in the reference databases it can be assumed that these new variants are possibly pathogenic. Their placement in the functional regions of the protein supports this conclusion.

Figure 3B indicates that p.N103K and p.M184T are localized on the prodomain region of the protein. The prodomain is cleaved during the maturation process and remains non-covalently bound to the homo or heterodimers upon secretion. The folding and dimerization of the mature protein are regulated by the prodomain [31]. It has been shown that prodomain mutations can prevent its binding to mature dimers leading to decreased BMP15 activity [32]. As mentioned previously, BMP15 reduction is correlated to increased ovulation rate and the chance of dizygotic twins’ pregnancy. This increase in ovulation can eventually lead to ovarian depletion and POI [33].

It should be noted that sheep *BMP15* mutations have often been occurred in the mature protein, while most human *BMP15* mutations are prodomain variants [26]. Nonsynonymous missense variants of *BMP15* that are listed in Table 3 are prodomain alterations except for; p.R329C, p.R329H and p.C357Y which are located on the mature TGFβ-like domain.

In 2018, Zhang et al. reported homozygous p.C357Y mutation in the TGFβ-like domain. To our knowledge, this is the sole described homozygous nonsynonymous missense variant of the *BMP15*. The patient`s heterozygous mother was 48 years old with normal menstrual cycles. They assumed that the mutations in the TGFβ-like domain should be homozygous to cause the disease while the heterozygous mutations of the prodomain are sufficient for defective protein maturation and POI [27]. However, Patiño et al. reported another mutation in TGFβ-like domain; p.R329H, in a heterozygous POI patient [26]. The p.R329C mutation of the mature subunit was reported earlier from China. The patient was a 37-year-old woman who developed secondary amenorrhea at the age of 27. The patient’s mother had irregular menses and menopause at the age of 40. The data on hetero or homozygosity of these mother and daughter is not available [24]. More recently, Rossetti et al. examined p.R329C mutation, functionally. The results showed that heterozygote p.R329C mutation led to disrupted co-localization with GDF9 and SMAD pathway activation [34].

We found p.N103K and p.M184T in the heterozygous patients, like almost all previously reported *BMP15* mutations [23]. This feature was often concluded by haploinsufficiency or negative dominance effects. However, the discovery of intragenic duplication of the TP63 in the carriers of p.Y235C has added to the complexity of interpreting the BMP15 mutations.

## Conclusions

In this study, we found two new variants in the *BMP15* gene that have not been reported in databases. These mutations were not identified in 800 Iranians whole-exome sequencing results available on Iranom’s website.

The p.N103K and p.M184T are localized on the prodomain region of the protein. Due to the cleavage of prodomain during BMP15 maturation, bioinformatics analyzes are likely to underestimate the effects of the mutations. This is while prodomain is actively involved in the homo and heterodimers secretion and function. Further studies to elucidate the roles of the prodomain could be of great value in identifying the effects of multiple mutations reported in this region.

## Data Availability

The authors confirm that the data supporting the findings of this study are available within the article. Raw data of are available from the corresponding author on request.
